# Crystal Structure of Human Soluble Adenylate Cyclase Reveals a Distinct, Highly Flexible Allosteric Bicarbonate Binding Pocket

**DOI:** 10.1002/cmdc.201300480

**Published:** 2014-02-24

**Authors:** Susanne M Saalau-Bethell, Valerio Berdini, Anne Cleasby, Miles Congreve, Joseph E Coyle, Victoria Lock, Christopher W Murray, M Alistair O'Brien, Sharna J Rich, Tracey Sambrook, Mladen Vinkovic, Jeff R Yon, Harren Jhoti

**Affiliations:** [a] Astex PharmaceuticalsCambridge Science Park, Cambridge, CB4 0QA (UK) E-mail: harren.jhoti@astx.com

**Keywords:** allosterism, drug discovery, enzyme regulation, fragment screening, structural biology

## Abstract

Soluble adenylate cyclases catalyse the synthesis of the second messenger cAMP through the cyclisation of ATP and are the only known enzymes to be directly activated by bicarbonate. Here, we report the first crystal structure of the human enzyme that reveals a pseudosymmetrical arrangement of two catalytic domains to produce a single competent active site and a novel discrete bicarbonate binding pocket. Crystal structures of the apo protein, the protein in complex with α,β-methylene adenosine 5′-triphosphate (AMPCPP) and calcium, with the allosteric activator bicarbonate, and also with a number of inhibitors identified using fragment screening, all show a flexible active site that undergoes significant conformational changes on binding of ligands. The resulting nanomolar-potent inhibitors that were developed bind at both the substrate binding pocket and the allosteric site, and can be used as chemical probes to further elucidate the function of this protein.

## Introduction

Bicarbonate (hydrogen carbonate) is biochemically indispensable to most life forms. It is continually produced in cells as a waste product of respiratory oxidation and plays an essential role in diverse biological processes ranging from photosynthesis, cellular and whole-body pH regulation, smooth and cardiac muscle contraction and relaxation, to signal transduction.[1] In mammals, bicarbonate regulates the concentration of the prototypical second messenger 3′,5′cyclic adenosine monophosphate (cAMP) via the stimulation of soluble adenylate cyclases (EC 4.6.1.1).[2–4] cAMP is solely produced by adenylate or adenylyl cyclases (AC), and its intracellular levels are tightly regulated. Mammalian cells express two different types of adenylate cyclases: the well-known transmembrane ACs (tmAC) and the more recently discovered soluble adenylate cyclase (solAC).[5] TmACs are a family of nine isoforms of plasma-membrane-bound proteins stimulated by hormones or neurotransmitters and activated by the α-subunit of the G_s_ protein. Each member’s differential response to G_i_, G_o_ and G_z_ α-subunit, G_z_ and Ca^2+^/calmodulin, allows strictly controlled intercellular signaling to occur within the appropriate cellular context.[6, 7] Soluble AC (solAC) is found in the cytosol, is structurally and biochemically distinct from the tmAC enzyme family, and is synergistically regulated by calcium and bicarbonate ions.[8] SolAC works in conjunction with phosphodiesterases, protein kinase A (PKA) and PKA- anchoring proteins (AKAP), as well as other effectors to establish and maintain signal transduction pathways. The original hypothesis that cAMP could only be produced at the cellular membrane by tmACs and would then diffuse inside the cell to effect specific cellular responses has, therefore, been superseded by a more comprehensive pathway and a model that postulates cAMP-specific microdomains.[7, 9, 10]

Human solAC (hsolAC) is encoded by a single gene, *ADCY10*, which produces two different-length splice variants: the minimal functional 48 kD variant contains solely the two heterologous catalytic domains, C1 and C2, whilst the full-length ∼190 kD variant comprises additional putative regulatory domains at the C terminus.[11–13] Both forms are fully active and synergistically stimulated by bicarbonate and calcium ions. solAC was first detected in testes and spermatozoa, and its role was thought to be confined to fertility and capacitation, an idea that appeared to be supported by solAC knockout mice, which appeared fully viable except for their inability to reproduce.[14–16] Consequently, solAC was considered a potential target for male contraception and a candidate for drug discovery efforts. Later, solAC was found to be present, albeit at lower concentrations, in most organs[10] and to be indispensable for a wide range of intracellular signaling events. Here, we describe the first crystal structures of a mammalian solAC, and the application of fragment-based drug discovery methods to identify small-molecule inhibitors of the human enzyme.

## Results

### Overall structure and active site

**Figure 1:**
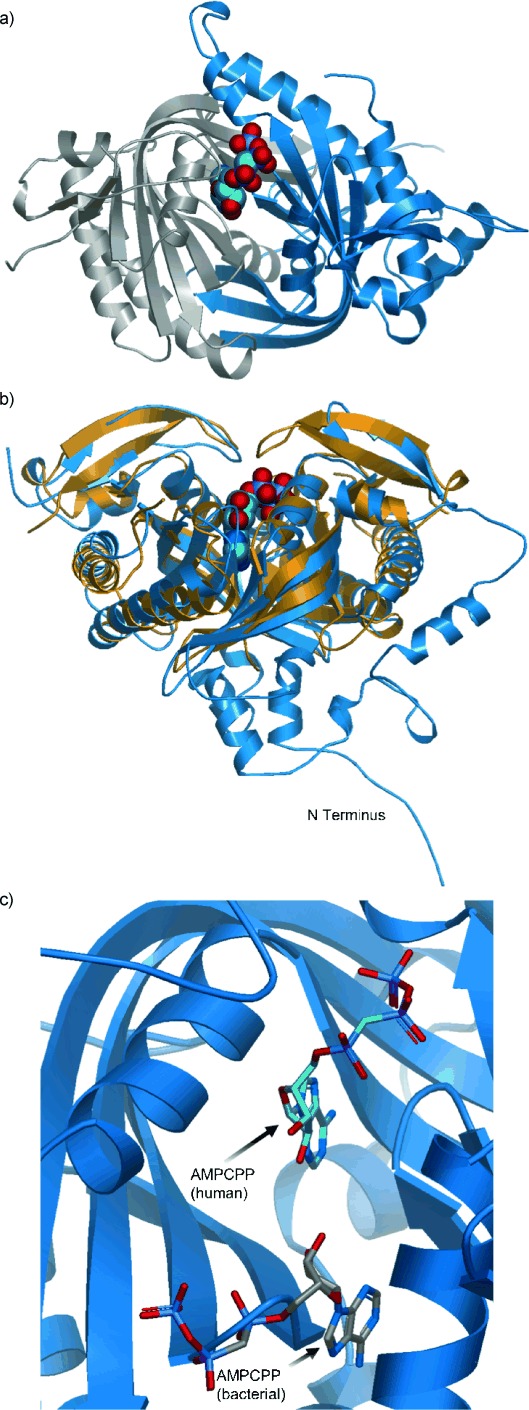
Crystal structure of the human adenylate cyclase. a) Ribbon diagram of the human soluble adenylate cyclase enzyme in complex with the substrate analogue AMPCPP (solid spheres). Domains C1 and C2 of the single chain are coloured blue and grey, respectively, and show the twofold pseudosymmetry of the protein. b) Overlay of the ribbon diagrams of the hsolAC–AMPCPP complex (blue) and the cyanobacterial solAC homodimer from *Spirulina platensis* (gold) showing the N-terminus and interdomain extensions of the human enzyme. The bound AMPCPP in hsolAC illustrates the location of the single active site. c) View of the hsolAC active site with bound AMPCPP (blue), with a molecule of AMPCPP (grey) from the *S. platensis* complex (PDB: 1WC0[17]) superimposed on the putative second site. The ribbon diagram of the human structure shows how the extension of β-strands 2′ and 3′ and the loop linking them (residues Met 337–Gly 341) occludes the site, obstructing the binding of a second nucleotide.

We determined the crystal structure of the short-form hsolAC at 1.7 Å resolution and show that the two domains, C1 and C2, adopt an approximate twofold pseudo-symmetrical conformation with the sole active site positioned off-center within the interdomain cleft (Figure 1 a; see also figure S1 in the Supporting Information). Comparison of the hsolAC structure with that of the previously determined orthologue from the cyanobacterium *Spirulina platensis* shows the secondary structural elements to be similar, though the external loops exhibit significant divergence and are generally longer in hsolAC (Figure 1 b).[17] These loops consist of N-terminal residues 1–28 and the interdomain residues 219–285, which form a subdomain of loops and helices that packs against the catalytic core of the enzyme. The human enzyme also displays a significant degree of mobility, and very weak main-chain density was seen for amino acids 135–140 and 350–356, making it hard to interpret.

Furthermore, while in the cyanobacterial enzyme there are two identical nucleotide binding sites, in the hsolAC structure only one of these sites is accessible because an extension of β strands 2 and 3 in C2 and the loop linking them precludes the binding of a second substrate molecule (Figure 1 c; see also figure S2 in the Supporting Information).

**Figure 2:**
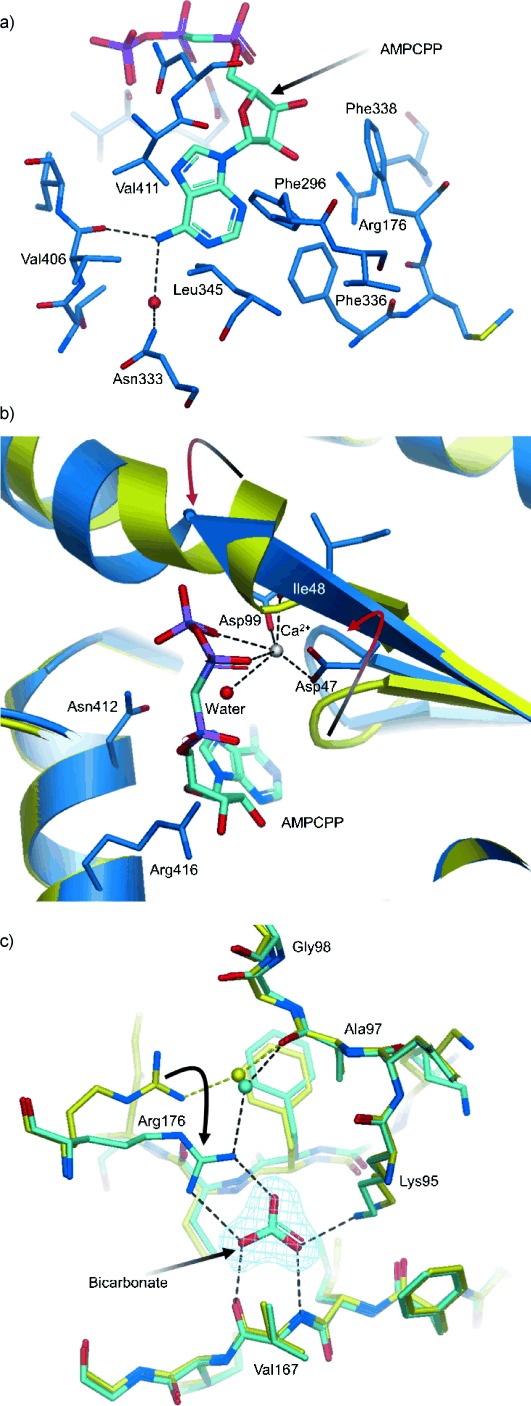
Close up views of the nucleotide and bicarbonate binding sites. a) hsolAC active site in complex with AMPCPP. The adenine pocket is shaped by hydrophobic residues Val 411, Phe 336, Leu 345, Phe 296 (Ala 97 is towards the back and not shown). The hydrogen bonds of the N6 amine to Val 406 backbone carbonyl and a water molecule (red sphere) are represented by dashed lines. Phe 338 and Arg 176 flank the ribose ring. b) Comparison between the apo (yellow) and AMPCPP–Ca^2+^ complex (blue) structures of hsolAC. The highly conserved Asn 412 and Arg 416 of the NXXXR cyclase catalytic motif hydrogen bond to the ribose and the phosphate oxygens. The Ca^2+^ ion (silver sphere) exhibits an octahedral coordination with β,γ oxygen atoms of the AMPCPP phosphates, a carboxyl oxygen each from the invariant Asp 47 and Asp 99 residues, the backbone carbonyl of Ile 48 and a water molecule (red sphere). The arrows highlight the movement of the β-hairpin loop and the unwinding of helix α3. c) Comparison of the apo (yellow) and bicarbonate-bound (cyan) hsolAC structures. Density for bicarbonate is shown (blue mesh). The bicarbonate anion forms hydrogen bonds (black dashed lines) with the backbone carbonyl and NH groups of Val 167, the guanidino group of Arg 176 and amine side chain of Lys 95. The side chain of Arg 176 undergoes an approximate 30° swing to hydrogen bond with bicarbonate, altering the water-mediated interaction with Ala 97.

Indeed, the crystal structure of the ternary complex of hsolAC with the substrate analogue α,β-methylene adenosine 5′-triphosphate (AMPCPP) and Ca^2+^ ion confirms this single ATP binding site. The adenine group of AMPCPP sits close to the bottom of a predominantly hydrophobic pocket formed by the side chains of Ala 97, Phe 296, Leu 345, Phe 336 and Val 411 (Figure 2 a). The N6 amine forms hydrogen bonds to the backbone carbonyl of Val 406 and to a water molecule located at the very bottom of the pocket. The environment of the ribose moiety is more amphipathic in nature, with the ring abutting the hydrophobic side of the pocket and making van der Waals contact with the side chains of Ala 415 and Phe 338. The guanidino group of Arg 176 points into the hydrophobic interior of the pocket, towards the 2’ and 3′ hydroxy groups of the ribose ring. Further along the pocket, the highly conserved Asn 412 and Arg 416 of the NXXXR cyclase catalytic motif straddle the ribose ring and the α phosphate (Figure 2 b). Both of these residues have been implicated in the stabilization of the transition state.[18] In our structure, the guanidino group of Arg 416 forms a hydrogen bond with the oxygen atoms from the α-phosphate, while the 2 amino group of Asn 412 hydrogen bonds to the oxygen atoms on the γ-phosphate. The Ca^2+^ ion interacts with the β,γ phosphates of AMPCPP, the backbone carbonyl of Ile 48, carboxylate oxygen atoms from the strictly conserved Asp 47 and Asp 99 amino acids and a water molecule, which in turn mediates an interaction with the α phosphate. Adenylate cyclases are known to require two metal ions to catalyse the phosphoryl transfer reaction. The observed Ca^2+^ ion in our structure occupies metal site B, but no discernible density was observed for the second metal site (A), which is also expected to be in close vicinity to the substrate.[19] Calcium is known to activate hsolAC by lowering the *K_m_* value for ATP–Mg^2+^ from about 10 mm to 0.9 mm.[8] The reduction in the *K_m_* value for ATP suggests that the calcium-coordinated phosphates contribute a large part of the binding energy for ATP. Comparison of the complexes of hsolAC and *S. platensis* solAC with AMPCPP/Ca^2+^ show that in both cases, the nucleotide analogues assume very similar productive conformations and are poised for the subsequent in-line reaction.[17] Binding of the nucleotide analogue AMPCPP/Ca^2+^ to hsolAC causes two major conformational changes in the active site pocket. Firstly, the N terminus of helix α3 unwinds by one turn upon formation of the complex-extending strand β1; and second, the β-hairpin loop from Ala 97 to Ala 100 moves by approximately 4 Å, presumably to accommodate substrate binding (Figure 2 b). The absence of a *S. platensis* solAC apo structure precludes such comparison; however, movements of the corresponding α-helix (α1) and β-hairpin loop (β7-β8) were ascribed to the binding of HCO_3_^−^ to the binary-complex crystals during flash soaking and freezing experiments.[17]

### Bicarbonate binding site

As previously indicated, in contrast to the cyanobacterium *S. platensis* protein, human solAC only binds one substrate molecule of AMPCPP as the putative second substrate binding site is significantly remodelled due to insertions. However, after soaking apo crystals of hsolAC with bicarbonate, we did observe additional electron density in this region, which is consistent with a bound bicarbonate ion (Figure 2 c). The bicarbonate anion appears to be tightly held in place by hydrogen bonds with the side chain amino group of Lys 95, the backbone amide and carbonyl groups of Val 167, the backbone amide of Met 337 and both terminal nitrogen atoms of the guanidino group of Arg 176. The side chain of Arg 176, which in the apo structure forms a water-mediated interaction with the backbone carbonyl of Ala 97, undergoes an approximate 30° swing in order to bind the bicarbonate ion disrupting the hydrogen bond to Ala 97 (Figure 2 c). Since this loop is located very close to the adenine of the substrate AMPCPP and contains one of the catalytically essential aspartic acid residues (Asp 99), the conformational flexibility observed by the binding of the bicarbonate ion and propagated via the movement of Arg 176 could influence the mechanism by which the bicarbonate ion exerts its allosteric regulatory role. Further evidence of the functional importance of one of these residues involved in the binding of bicarbonate, Lys 95, is provided by its conservation across orthologous enzymes from species including cyanobacterial *Anabaena sp.* PCC7120, *S. platensis* and rat solAC.[20]

### Fragment screening and inhibitor design

**Figure 3:**
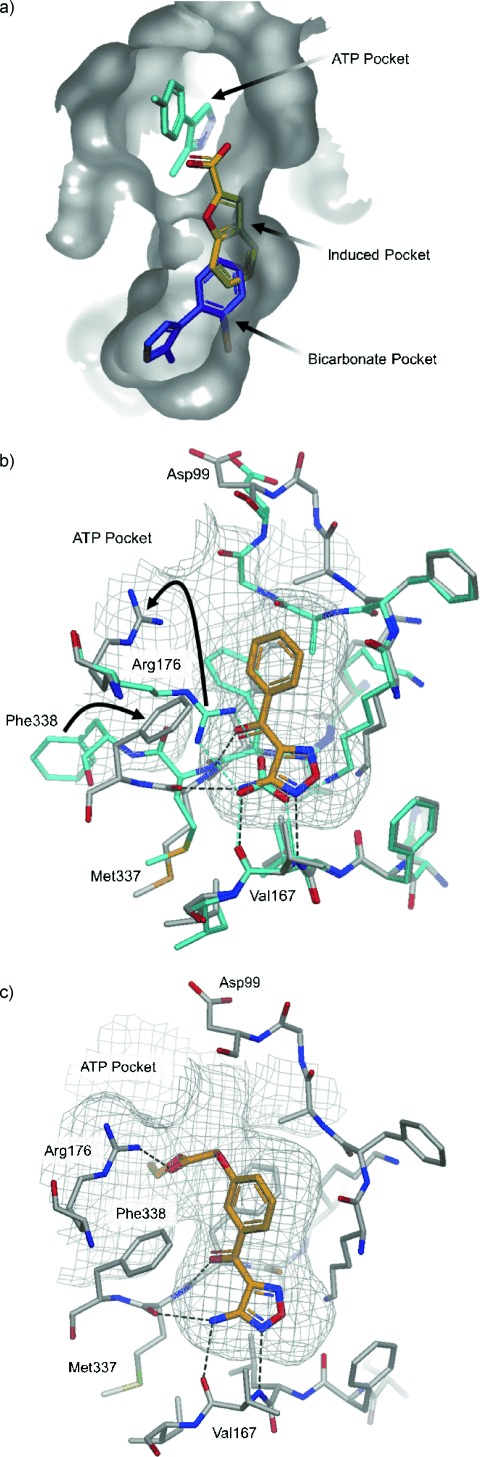
Fragment binding and structure-guided compound optimisation. a) Overlay of three different crystal structures showing fragments binding in multiple regions of the hsolAC catalytic and allosteric pockets. The phenyl pyrazole (cyan) binds in the ATP pocket, an aromatic carboxylic acid (2; yellow) induces a new pocket and another pyrazole (3; magenta) binds in the allosteric bicarbonate pocket. b) Overlay of the bicarbonate (cyan) and amino-furazane fragment (4; orange) protein structures. Both molecules interact with the Val 167 backbone. Compound 4 triggers movement of the side chains of Arg 176 (close to the position the residue adopts in the apo structure) and Phe 338, altering the shape and electrostatic environment of the pocket. The ketone group of compound 4 forms a hydrogen bond with the backbone NH of Met 337, whilst the carbonyl of Met 337 forms an additional hydrogen bond with compound 4. The phenyl ring of the fragment sits in a hydrophobic pocket between Phe 45 and Phe 336 (not labelled, shown faded on the back). Position 3 on the phenyl ring of compound 4 presents a good vector to access the ATP pocket. c) Complex of hsolAC with compound 7 (orange). The ethyl ester added to the 3-methoxy group of compound 6 exploits the movement of Arg 176 observed with compound 4 (panel b) forming an additional hydrogen bond with its side chain and shows opportunity to further extend the inhibitors into the ATP pocket. d) Overlay of the protein complex of hsolAC with compounds 4 (cyan) and 8 (orange) showing the growth of the molecule from the original fragment. Interactions with both Val 167 and Met 337 backbones are preserved along the series. The molecule extends towards the ATP pocket forming two further hydrogen bonds: one with Arg 176 (also seen in the complex with compound 7) and an additional one, accessible through the movement of the Asp 99 side chain.

To identify inhibitors of human solAC, a library of 1600 fragments was screened using thermal shift (*T_m_*), functional bioassays, and X-ray crystallography. The crystal structures of the protein–fragment complexes revealed high intrinsic flexibility in the protein, as binding of these relatively small and low-affinity compounds was able to alter the shapes of both the active and allosteric sites. The majority of the fragments bound in the bicarbonate binding site and represented carboxylic acids and acidic heterocycles binding in their anionic form. However, three fragment hits were also found to bind in the ATP binding site, and a further two fragments bound in an induced pocket located between the ATP and bicarbonate binding sites (Figure 3 a and Table 1). Initial fragment hits were followed up with analogue searches from an in-house repository, based on either substructure derivatives and/or *iso*-structural heterocycles. These were ranked based on their affinity in a functional bioassay and then soaked into hsolAC crystals to obtain additional protein–fragment crystal structures. The most potent inhibitors from this round of screening were found to be amino-furazanes (e.g., **4**; IC_50_=19 μm) and trifluoro-pyrazoles (e.g., **5**; IC_50_=28 μm), both of which bound in the bicarbonate binding site.

**Table 1:** In vitro activity profiles of compounds binding to hsolAC

Compd	Structure	CLog *P*	IC_50_ [μm]^[a]^	LE^[b]^
**1**	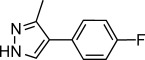	2.3	>1000	–
**2**	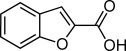	2.4	>1000	–
**3**	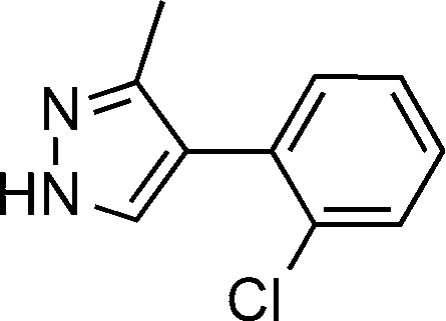	2.6	>300	–
**4**	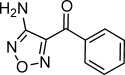	2.5	19	0.46
**5**	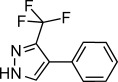	3.4	28	0.41
**6**	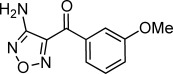	2.4	11	0.42
**7**	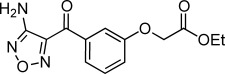	2.6	1.6	0.38
**8**	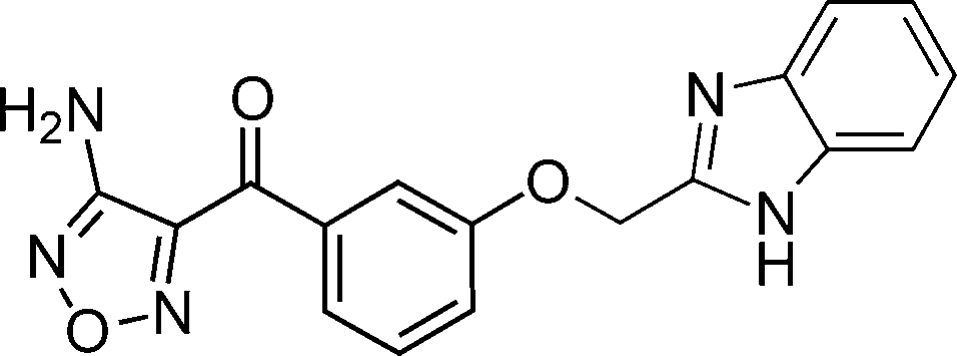	3.6	0.36	0.35

[a] The half maximal inhibitory concentration (IC_50_) of the compounds was measured in the bioassay as described in the Experimental Section. Assay conditions were optimized for maximum signal, and all compounds except for compound **7** were independently tested at least twice in duplicate with SD in line with **5**, the control compound. Compound **5** was used as the assay standard (*n*=19 independent experiments; data is the geometric mean; SD=±3.8 μm). [b] Ligand efficiency (LE) is defined as the binding affinity per heavy atom and has units of kcal mol^−1^ *N*^−1^, where *N* is the number of non-hydrogen atoms.

To select which fragment hits to develop into inhibitors, we evaluated the hits on the basis of binding interactions, novelty, synthetic accessibility, as well as affinity. The amino-furazanes (e.g., **4**) were the most potent compounds identified from the follow-up of the initial hits. Their unequivocal binding mode in the bicarbonate pocket, noncharged (neutral) state, and well-positioned vectors extending towards the ATP site (Figure 3 b), fulfilled most of the criteria required to develop these into a lead series. The amino-furazane heterocycle, a constant scaffold in the whole series, mimics the donor/acceptor hydrogen bonding pattern of the bicarbonate with the Val 167 backbone. The molecule also forms two further hydrogen bonds to the carbonyl (C=O) and backbone NH of Met 337. The absence of a negative charge, as in the bicarbonate anion, is likely responsible for the rearrangement of the positively charged Arg 176 side chain, which moves out of the pocket towards the solvent-exposed entrance and is replaced by the side chain of Phe 338, creating a more hydrophobic environment. The phenyl ring of the fragment sits in a narrow hydrophobic cleft flanked by Phe 45, Phe 336 and Phe 338, with the latter two residues undergoing substantial conformational changes induced by fragment binding.

The basic strategy in optimizing fragment **4** was to grow from the bicarbonate pocket through the induced pocket region that lies adjacent to Phe 336 and into the ATP pocket. Initially, a number of simple linker groups were attached onto the phenyl of fragment **4**, and the 3-methoxy analogue, **6**, proved to be the most promising with similar potency and excellent growth vectors (**6**; IC_50_=11 μm). The next round of synthesis focused on molecules capable of forming a hydrogen bond with the side chain of Arg 176; this was an attractive design idea because the side chain was easily accessible and sits at the mouth of the ATP pocket. A simple ethyl ester attachment to the methoxy group resulted in a 10-fold improvement in potency (**7**; IC_50_=1.6 μm), and the crystal structure of the co-complex is consistent with the formation of the targeted hydrogen bond (Figure 3 c). A number of heterocycles were then synthesized that could retain this hydrogen bond whilst improving the π-stacking with Phe 336. This culminated in the discovery of the novel lead compound **8** (IC_50_=0.36 μm). The designed molecule induces a movement of the side chain of Asp 99 to form an additional long hydrogen bond with the benzimidazole of compound **8** (Figure 3 d). The molecule also forms a hydrogen bond with the flexible side chain of Arg 176, although the geometry is not ideal. The fragment-to-lead campaign outlined here illustrates two important points: firstly, the binding mode of the starting fragment, **6**, was recapitulated in the final molecule (Figure 3 d); and secondly, the fragment- induced movement of the Phe 338 and Arg 176 side chains could be exploited via careful structure-based optimisation.

## Discussion

The hsolAC crystal structure provides the first description on a molecular basis of an enzyme that is functionally regulated by a bicarbonate ion. Moreover, the structure highlights how the human protein has substantially diverged from its cyanobacterial orthologue. Most notably, there is a single binding site for the substrate ATP, with the protein symmetry-related site (equivalent to the second ATP site in the bacterial enzyme) now able to bind the allosteric activator bicarbonate. It is therefore clear that the fusion event that created the single chain C1/C2 mammalian enzyme[21] has allowed differences to evolve in catalytic mechanism and regulatory control compared with the homodimeric bacterial orthologues. Intriguingly, the crystal structures of mammalian tmACs also show a similar C1/C2 architecture—a single active site located at the heterodimer interface and a pseudo-symmetrical, catalytically incompetent site, where the nonphysiological forskolin activator is seen to bind.[19, 22, 23]

Bicarbonate acts on mammalian solACs both to relieve substrate inhibition and to increase the maximum reaction velocity (*V*_max_).[8] The crystal structures presented here show how the allosteric site is exquisitely designed to recognise the bicarbonate ion, which is held by six hydrogen bonds including a hydrogen bond to the backbone carbonyl of Val 167, which allows the protein to discriminate between HCO_3_^−^ and alternative anions that are unable to act as hydrogen-bond donors. The structures also suggest that bicarbonate exerts its regulatory effect via an allosteric mechanism, in which conformational changes are propagated to the substrate site via Arg 176 (Figure 2 c). One of the key residues involved in bicarbonate binding, Lys 95, has also been shown to be important in the bacterial orthologue from *Anabaena*; site-directed mutagenesis of the analogous residue and subsequent enzyme kinetic studies confirmed its crucial role for activation of the enzyme by bicarbonate.[18] However, given the fact that the other residues involved in bicarbonate binding in the hsolAC structure are not conserved in the bacterial orthologues, the precise mechanism by which bicarbonate regulates these proteins remains unclear.

The paper also describes the first structurally characterised potent inhibitors of hsolAC. The inhibitors were discovered by fragment-based drug discovery and occupy both the newly identified allosteric bicarbonate site of the protein as well as the ATP pocket. The work provides further support for the potential of fragment-based screening to identify and exploit novel allosteric pockets for inhibitor design.[24–27] Alternative protein conformations and loop movements are difficult to predict, and the energetics of such movements could mask the binding of low-affinity fragments. However, we observed several fragment molecules binding at pre-existing sites, whilst others caused significant loop movements and induced new additional binding pockets. Major conformational movement induced by the binding of low-affinity fragments has been reported previously and indicates that, while the affinity of fragments may be low, their binding efficiency can be significant.[28] Indeed, we were able to elaborate the original low-affinity fragment hits into potent lead compounds, and we believe that the iterative use of experimentally determined binding modes was important in achieving this goal.

In summary, the active site flexibility of hsolAC did not pose a barrier to fragment screening or to subsequent optimisation of binding potency, therefore, these approaches can be successful at targeting highly flexible proteins. The resulting compounds described here are not only potential drug candidates, but also potent chemical probes and might be useful for the further characterisation of the function of soluble adenylate cyclase in higher organisms.

## Experimental Section

### Protein expression and purification

*Cloning*: An expression vector pCDNA3.1 encoding full-length solAC (Nucleotide NM 018417 SwissProt) was used as a template for polymerase chain reaction (PCR) amplification. The construct M1V469 was amplified by PCR, using 3′ and 5′ primers.

5′ primer: 5′-GGGGACAAGTTTGTACAAAAAAGCAGGCTCCATGAACACTCCAAAAGAAGAATTCCAGGACTGG-3′, which carried an attB1 recognition sequence, and a sequence corresponding to bases 1–11.

3′ primer: 5′-GGGGACCACTTTGTACAAGAAAGCTGGGTTCAGTGGTGGTGGTGGTGGTGGACTTTCTCAGTACGGCCC-3′, which introduced a sequence corresponding to bases 463–469, a six his C-terminal tag, a stop codon, and an attB2 recognition site.

PCR reagents: Thermopol buffer 10X (10 μL), dNTP mix (2 μL), DNA template (1 μL), 5′ primer (1 μL), 3′ primer (1 μL), Vent (1 μL). Reaction made up to 100 μL with H_2_O.

PCR cycling: 25 cycles of 94 °C for 30 s, 55 °C 1 min, 72 °C for 3 min, followed by an extension of 10 min at 72 °C. The 1407 bp fragment was recombined into the entry vector, pDONR, using the BP reaction of the Gateway cloning technique. Product was transformed into DH5α competent cells; plasmid was extracted from the bacteria grown on the kanamycin selective plates. The sequence was confirmed by DNA sequencing before transferring the clone to the destination vector, pDEST8, via the LR reaction of the Gateway cloning technique. An aliquot of the product was transformed into DH5α competent cells. Plasmid was extracted from the bacteria grown on the carbenicillin-selective plates. The sequence was confirmed by DNA sequencing before beginning expression of the protein. Expression of the solAC constructs was done in High Five cells (BTI-TN-5B1-4).

*Expression of hsolAC*: Production of recombinant virus of hsolAC was performed in the following manner. Briefly, the pDEST8 vector encoding the relevant gene was transformed into *Escherichia coli* DH10BAC cells containing the baculovirus genome (bacmid DNA). Via a transposition event in the cells, a region of the pDEST8 vector containing the gene and a gentamycin-resistance gene including the baculovirus polyhedron promoter was transposed directly into the bacmid DNA. By selection on gentamycin, kanamycin, tetracycline and Bluo-gal, resultant white colonies should contain recombinant bacmid DNA encoding the relevant gene. Bacmid DNA was extracted from a culture of white DH10BAC cells and transfected into *Spodoptera frugiperda* sf9 cells grown in SF900 II serum-free medium following the manufacturer’s instructions. Virus particles were harvested 72 h post-infection. An aliquot (1 mL) of harvested virus particles was used to infect sf9 cells (100 mL) containing 1×10^6^ cells mL^−1^. Cell culture medium was harvested 72 h post-infection.

High Five insect cells were cultured in EX-Cell 405 (JRH) serum-free medium to a density of 1×10^6^ cells mL^−1^. An aliquot (5 mL) of viral stock was added to each litre of cells. The cultures were incubated at 27 °C for 48–72 h. Cells were harvested by centrifugation at 4000 rpm for 8 min 10 °C. Pellets were frozen at −80 °C.

*Purification of hsolAC*: All procedures were performed at 4 °C unless stated otherwise. Cell pellets were thawed on ice and re-suspended in lysis buffer [50 mm Tris (pH 7.5), 300 mm NaCl, 10 % glycerol, 2 mm β-mercaptoethanol (BME), protease inhibitor cocktail (Calbiochem)]. Cells were lysed by sonication, and lysate was incubated with DNase 1 for 1 h at 4 °C. Lysate was clarified by centrifugation at 14 000 or 25 000 rpm for 1 h. The clarified lysate was further clarified by centrifugation as in the previous step, then passed through a 0.45 μm filter before being applied to metal chelating matrix (GE Healthcare) pre-charged with Ni^2+^ in batch bind mode. The resin or matrix was poured into a column, and the solAC protein was eluted by addition of lysis buffer containing 250 mm imidazole. Fractions were analysed by sodium dodecyl sulfate–polyacrylamide gel electrophoresis (SDS–PAGE), and those containing solAC protein were pooled. Fractions containing hsolAC were tracked at each step by Western blotting using an hsolAC-specific monoclonal antibody (kindly supplied by Schering AG). The pooled protein was buffer-exchanged into low salt by application to a G25-desalting column equilibrated in 50 mm Tris (pH 7.5), 30 mm NaCl, 10 % glycerol, 5 mm BME. The buffer-exchanged solAC protein was then applied to a 6 mL Resource Q cation exchange column (GE Healthcare) and eluted using a gradient of 0–30 % 1 m NaCl over 20 column volumes. Fractions were analysed by SDS–PAGE and those containing solAC protein were pooled and applied to a 26/60 superdex-75 gel filtration column pre-equilibrated using 50 mm Tris (pH 7.5), 330 mm NaCl, 10 % glycerol, 5 mm BME. Fractions were analysed by SDS–PAGE. SolAC fractions were pooled and concentrated to a final concentration of ∼10 mg mL^−1^ using a vivaspin 2 centrifugal concentrator (HY membrane).

### Crystallization

Crystals of hsolAC were grown by the hanging-drop vapour diffusion method. Protein solution (1 μL) was mixed with 1 μL of reservoir solution (0.1 m sodium acetate (pH 4.8), 0.2 m trisodium citrate, 16–18 % PEG4K and 10 % glycerol) and left to equilibrate at 4 °C. Crystals appeared in the drops after 3–6 days and reached a maximum size of 0.5×0.1×0.1 mm in 14 days. Consistency of crystal size and quality was greatly improved by microseeding.

**Data collection and processing**: Crystals of solAC were briefly transferred to a cryobuffer solution (0.1 m sodium acetate (pH 4.8), 0.2 m trisodium citrate, 30 % PEG4K and 10 % glycerol) and plunged into liquid nitrogen prior to data collection at 100 K.

Datasets for hsolAC as well as for the heavy atom derivatives were collected in house using either a Jupiter CCD detector or an RAXIS HTC image plate detector. Both were mounted on Rigaku rotating anode generators. The high-resolution data used to refine the solAC structure at 1.7 Å was collected on Beamline ID29–1 at the European Synchrotron Radiation Facility (Grenoble, France), using an ADSC Quantum4 CCD detector, with a wavelength of 0.934 Å and processed using MOSFLM version 6.2.3.[29] The dataset was scaled using SCALA,[30] and the intensities converted to structure factor amplitudes with TRUNCATE.[31] The crystals grew in space group P6_3_ with cell dimensions of *a*=*b*=99.15 Å, *c*=97.51 Å.

**Structure determination and refinement**: The structure of hsolAC was solved by multiple isomorphous replacement using five heavy-atom-containing derivatives (table S1 in the Supporting Information). Difference Patterson methods were used to obtain initial positions for the heavy atoms. Preliminary phasing with MLPHARE,[30] generated difference Fourier maps that were then inspected for additional sites. Map quality was improved using solvent flattening (Solomon and cycles of ARP/WARP). The sequence of hsolAC was built into the resulting electron density map. After multiple rounds of rebuilding and refinement (using QUANTA, REFMAC and CNX) at 1.7 Å using the synchrotron data, the final soluble adenylate cyclase model consisted of residues 1–468, with two gaps (residues 135–140 and 350–356). The final refinement statistics are shown in table S2 in the Supporting Information.

**Preparation of hsolAC–AMPCPP co-crystals**: 200 mm α,β-methylene adenosine 5′-triphosphate (AMPCPP), 40 mm CaCl_2_ and 40 mm MnCl_2_ were dissolved in 0.1 m sodium acetate (pH 4.8), 0.2 m trisodium citrate, 16 % PEG4K, and 10 % glycerol. A previously grown crystal of hsolAC was placed in 20 μL of this soaking solution and allowed to equilibrate for 3 days. The crystal was then moved to a solution of cryoprotectant, frozen in liquid nitrogen, and X-ray data collected.

**Preparation of solAC–HCO_3_**^**−**^ **co-crystals**: 50 mm sodium bicarbonate was prepared in a solution consisting of 0.1 m sodium acetate (pH 4.8), 0.2 m trisodium citrate, 16 % PEG 4000, and 10 % glycerol. A previously grown crystal of the solAC catalytic domain was placed in 20 μL of the bicarbonate solution and allowed to equilibrate for 3 h. The crystal was then moved to a solution of cryoprotectant and X-ray data collected.

**Preparation of compound–solAC co-crystals**: Compounds were soaked into hsolAC crystals at a concentration of 25 mm, the crystals subsequently being frozen, and data collected (tables 1 a and 1 b in the Supporting Information).

**RCSB Protein Data Bank accession codes**: The atomic coordinates and structure factors for the apo and co-crystal complexes described here have been deposited in the RCSB Protein Data Bank with accession codes: 4OYW (apo hsolAC); 4OYX (hsolAC–AMPCPP–Ca^2+^); 4OYZ (hsolAC–HCO^3−^); 4OZ2 (hsolAC–compd **1**); 4OYP (hsolAC–compd **2**); 4OYO (hsolAC–compd **3**); 4OYI (hsolAC–compd **4**); 4OZ3 (hsolAC–compd **5**); 4OYM (hsolAC–compd **6**); 4OYB (hsolAC–compd **7**); 4OYA (hsolAC–compd **8**).

### Biological evaluation

*Bioassay*: The enzymatic activity of hsolAC was measured in 96-well (black) plate format using the Mediomics Bridge-It designer cAMP kit (cat #122 935, Mediomics, LLC, St. Louis, MO, USA). The assay reactions contained 50 mm Tris-HCl (pH 7.4), 3 mm MnCl_2_, 0.1 % BSA, 500 μm ATP, 62.5 pm hsolAC, and test compound, and allowed to proceed for 20 min at RT. The reaction was stopped by adding 5 µL of 15 mm EDTA. The Bridge-It assay reagents were prepared as per the manufacturer’s instructions. The Bridge-It read mix was incubated with the stopped reactions for 1 h at RT before the fluorescence signal was read at 535 nm on excitation at 485 nm. The results from the hsolAC curves were calibrated against a standard curve of cAMP in assay buffer. IC_50_ values were generated using Graphpad Prism software (version 3.02).

*Thermal shift assay*: Thermal shift assays were performed on an Mx3005P quantitative PCR instrument (Stratagene, La Jolla, California) capable of temperature control and fluorescence detection. Protein unfolding was monitored using the SYPRO orange dye (Invitrogen), which binds to hydrophobic regions on the protein during the unfolding process. Compound stock solution (1 or 5 μL) was aliquoted into 96-well PCR plates (Starlab) to produce final nominal concentrations of 1 mm and 5 mm compound in 1 or 5 % DMSO, respectively. The hsolAC protein concentration was 0.25 μm in a buffer containing 50 mm HEPES (pH 7.5), 100 mm NaCl, 1 mm MnCl_2_, 1 mm dithiothreitol, and 2.5X SYPRO orange. Control experiments were performed on each 96-well plate, with six negative and two positive control wells per plate. The plate was heated over a temperature range of 25–70 °C, and the temperature was increased by 1 °C per min. The fluorescence intensity was measured every 0.5 °C. All data were processed and analysed using Origin 7.0 software.

### Chemistry

**Scheme 1:**
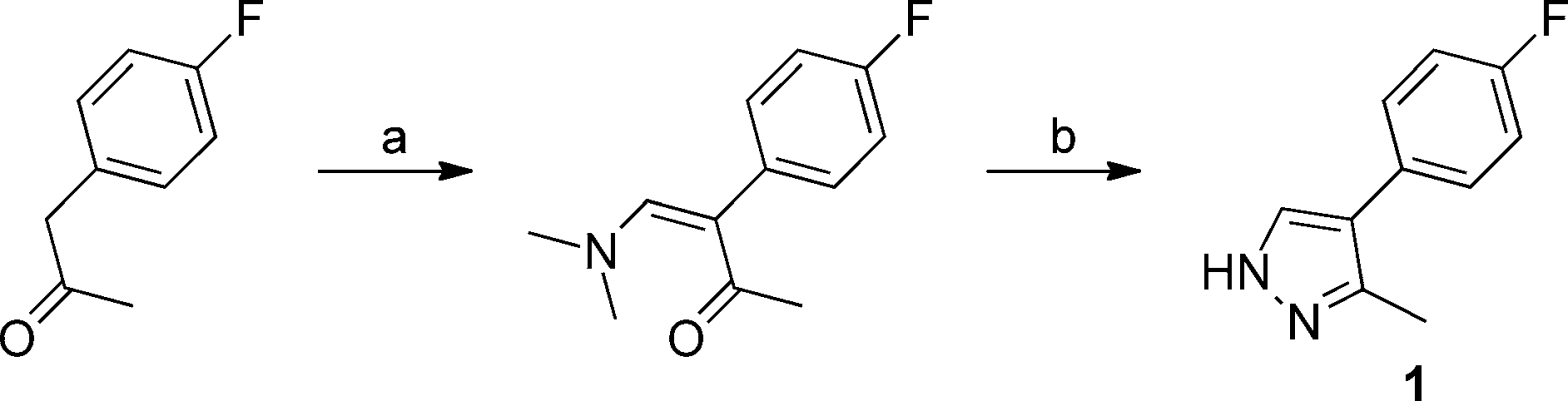
*Reagents and conditions*: a) DMF⋅DMA, toluene, reflux, 18 h; b) N_2_H_4_⋅H_2_O, EtOH, N_2_, reflux, 72 h.

**Scheme 2:**
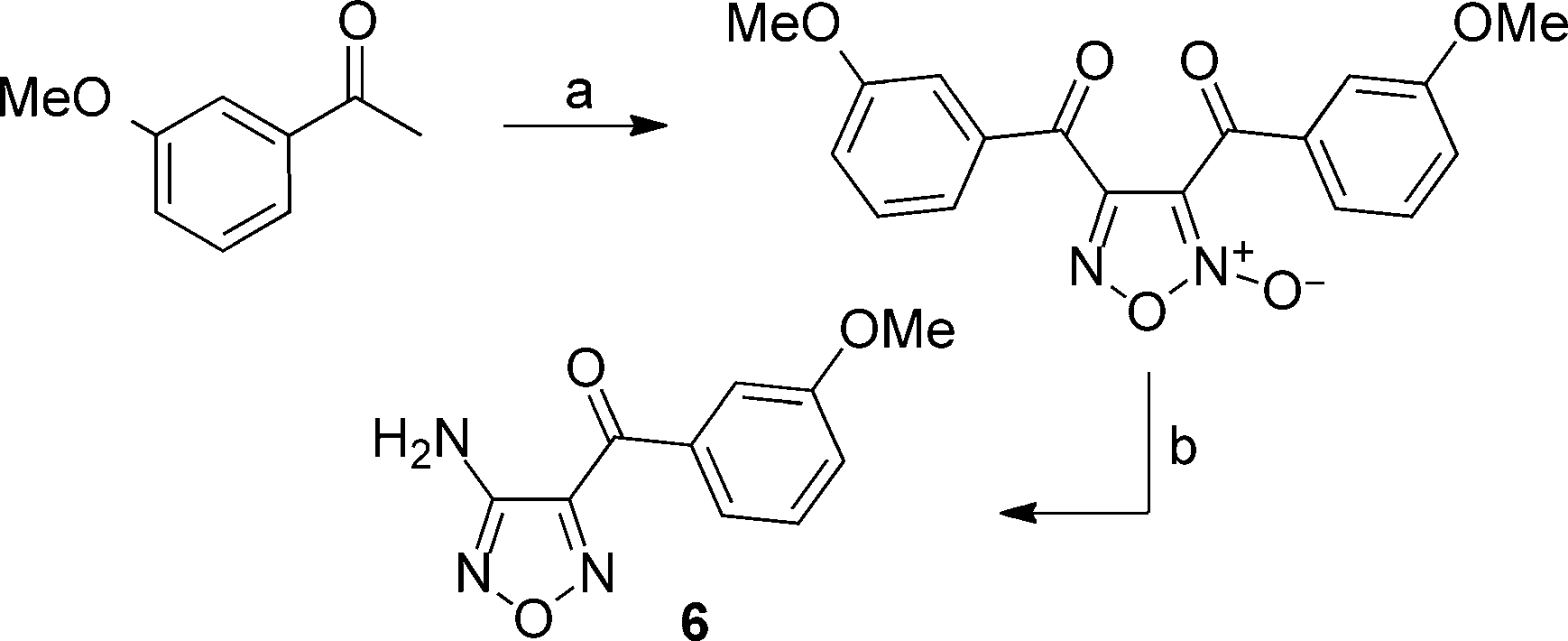
*Reagents and conditions*: a) HNO_3_, AcOH, NaNO_2_, 60 °C, 2 h; b) NH_3_, Et_2_O, MeOH, 60 °C, 2 h.

**Scheme 3:**
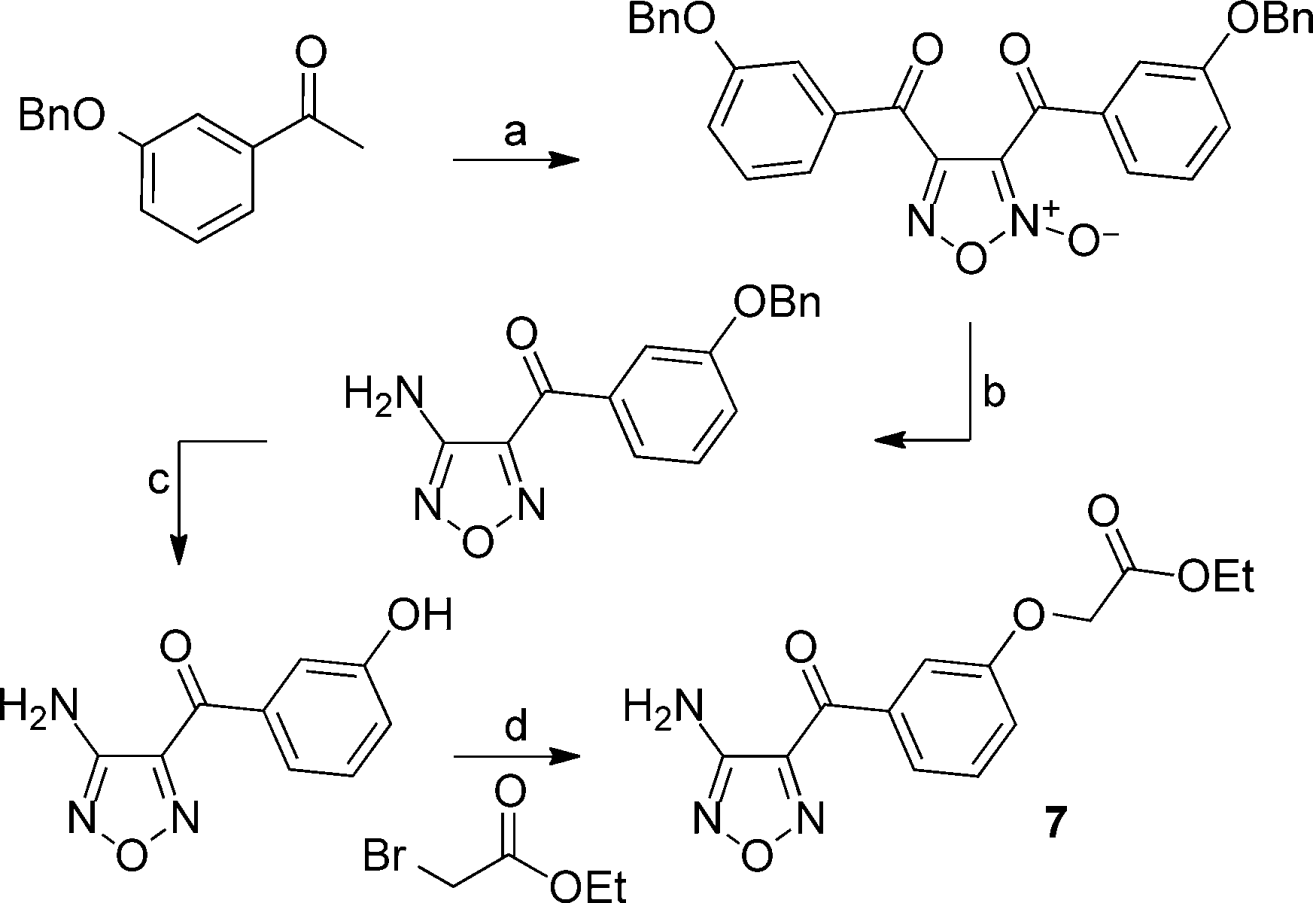
*Reagents and conditions*: a) HNO_3_, AcOH, NaNO_2_, 60 °C, 2 h; b) NH_3_, Et_2_O, MeOH, 60 °C, 2 h; c) BCl_3_, CH_2_Cl_2_, RT, 30 min; d) NaH, DMF, 90 °C, 2 h.

**Scheme 4:**
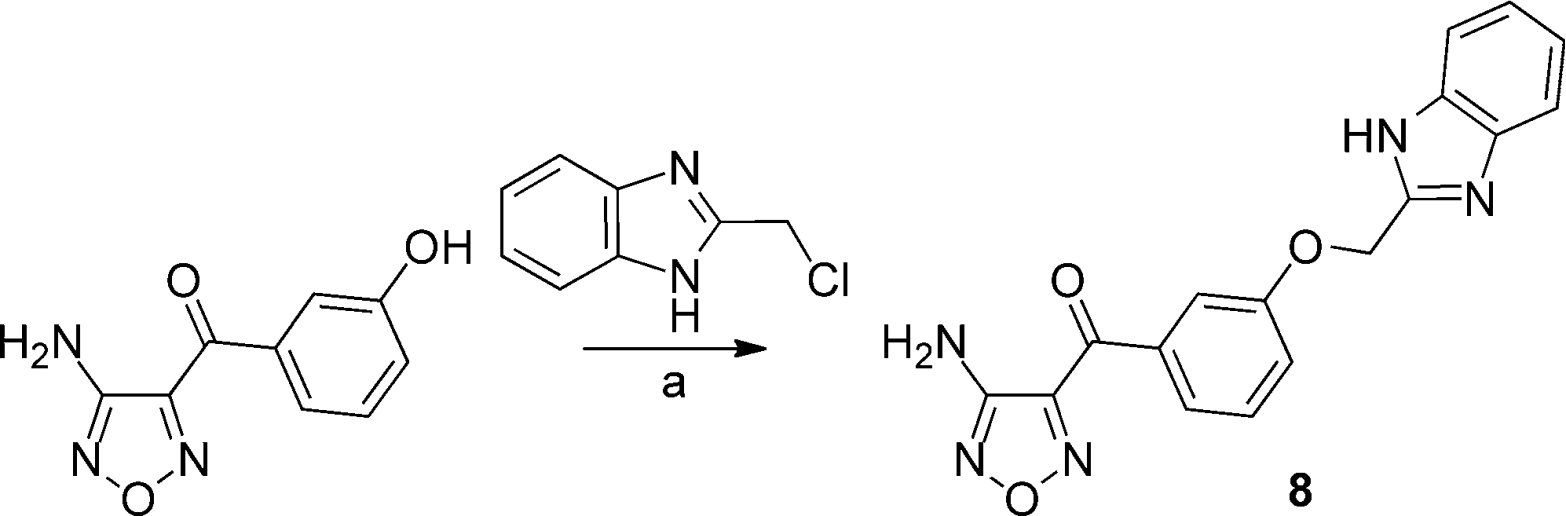
*Reagents and conditions*: a) NaH, DMF, 75 °C, o/n.

Compound **2** was purchased from Lancaster Synthesis. Compounds **3** and **5** were purchased from Maybridge. Compound **4** was purchased from Chemical Blocks. Compounds **1** and **6**–**8** were prepared as shown in Schemes 1–4 and described below.

**4-(4-Fluorophenyl)-3-methyl-1*H*-pyrazole (1)**: A mixture of 4-fluorophenylacetone (2.67 mL, 20 mmol) and *N*,*N*-dimethylformamide–dimethylacetal (4.0 mL, 30 mmol) in toluene (50 mL) was stirred at reflux for 18 h and then allowed to cool to RT. The solvent was removed in vacuo to afford (*Z*)-4-dimethylamino-3-(4-fluorophenyl)but-3-en-2-one as an orange solid, which was used without further purification (4.15 g, >100 %—likely due to solvent contamination): ^1^H NMR (400 MHz, [D_6_]DMSO): *δ*=7.51 (s, 1 H), 7.11 (m, 4 H), 2.67 (br s, 6 H), 1.97 ppm (s, 3 H); MS (ESI): *m*/*z*=208 [*M*+H]^+^.

A mixture of (*Z*)-4-dimethylamino-3-(4-fluorophenyl)but-3-en-2-one (4.1 g, 19.8 mmol) and NH_2_NH_2_⋅H_2_O (1.06 mL, 21.8 mmol) in EtOH (50 mL) was stirred at reflux under a nitrogen atmosphere for 72 h and then allowed to cool to RT. The solvent was removed in vacuo, and the residue was purified by recrystallization from petroleum ether and EtOAc. The resulting solid was collected by suction filtration, rinsed with petroleum ether, and dried in vacuo to afford **1** as an off-white solid (2.78 g, 79.7 %): ^1^H NMR (400 MHz, [D_6_]DMSO): *δ*=12.65 (br s, 1 H), 7.71 (br s, 1 H), 7.46 (m, 2 H), 7.21 (t, 2 H), 2.35 ppm (s, 3 H); MS (ESI): *m*/*z*=177 [*M*+H]^+^.

**(4-Amino-furazan-3-yl)-(3-methoxy-phenyl)-methanone (6)**: A mixture of 3-methoxyacetophenone (10 mL, 75.4 mmol), HNO_3_ (13 mL), AcOH (60 mL) and water (40 mL) was heated to 60 °C while stirring. A small quantity of NaNO_2_ (a spatula full) was added, and the mixture stirred at 60 °C for a further 2 h. The mixture was allowed to cool to RT and then partitioned between 2 m aq NaOH and EtOAc. The organic layer was separated, dried over anhydrous MgSO_4_, filtered and concentrated in vacuo to afford a viscous orange oil. Purification by column chromatography on silica gel (EtOAc/petroleum ether, 0–40 %) afforded [4-(3-methoxybenzoyl)-2-oxy-furazan-3-yl]-(3-methoxyphenyl)methanone as a viscous yellow oil (12.2 g, 89.9 %): ^1^H NMR (400 MHz, [D_6_]DMSO): *δ*=7.74 (dm, 1 H), 7.59 (m, 1 H), 7.54 (m, 2 H), 7.45 (m, 2 H), 7.38 (dm, 1 H), 7.30 (dm, 1 H), 3.83 (s, 3 H), 3.77 ppm (s, 3 H); MS (ESI): *m*/*z*=208 [*M*+H]^+^.

2 m NH_3_ in MeOH (60 mL) was added to a solution of [4-(3-methoxybenzoyl)-2-oxyfurazan-3-yl]-(3-methoxyphenyl)methanone (6 g, 13.3 mmol) in Et_2_O (30 mL) and MeOH (60 mL), and the mixture was stirred at RT for 30 min then heated to 60 °C for 2 h. Upon cooling to RT, the mixture was evaporated to dryness in vacuo, and the residue was purified by column chromatography on silica gel (EtOAc/petroleum ether, 0–30 %) to give **6** as an off-white solid (1.1 g, 37.8 %): ^1^H NMR (400 MHz, [D_6_]DMSO): *δ*=7.77 (dm, 1 H), 7.65 (m, 1 H), 7.55 (t, 1 H), 7.35 (dm, 1 H), 6.60 (br s, 2 H), 3.85 ppm (s, 3 H).

**[3-(4-Aminofurazan-3-carbonyl)phenoxy]acetic acid ethyl ester (7)**: A mixture of 3-benzyloxyacetophenone (15 g, 66.4 mmol), HNO_3_ (11.5 mL), AcOH (52 mL) and water (35 mL) was heated to 60 °C while stirring. A small quantity of NaNO_2_ (a spatula full) was added, and the mixture stirred at 60 °C for a further 2 h. The mixture was allowed to cool to RT and then partitioned between 2 m aq NaOH and EtOAc. The organic layer was separated, dried over anhydrous MgSO_4_, filtered and concentrated in vacuo to afford a viscous orange oil. Purification by column chromatography on silica gel (EtOAc/petroleum ether, 0–35 %) afforded [4-(3-benzyloxybenzoyl)-2-oxy-furazan-3-yl]-(3-benzyloxyphenyl)methanone as a yellow oil (9.3 g, 55.4 %): ^1^H NMR (400 MHz, [D_6_]DMSO): *δ*=7.76 (m, 2 H), 7.58 (m, 3 H), 7.50–7.30 (m, 13 H), 5.16 (s, 2 H), 5.08 ppm (s, 2 H).

2 m NH_3_ in MeOH (18 mL) was added to a solution of [4-(3-benzyloxybenzoyl)-2-oxyfurazan-3-yl]-(3-benzyloxyphenyl)methanone (9.3 g, 18.4 mmol) in Et_2_O (30 mL) and MeOH (60 mL), and the mixture was stirred at RT for 30 min then heated to 60 °C for 2 h. Upon cooling to RT, the mixture was evaporated to dryness in vacuo, and the residue was purified by column chromatography on silica gel (EtOAc/petroleum ether, 0–50 %) to give (4-aminofurazan-3-yl)-(3-benzyloxyphenyl)methanone as an off-white solid (920 mg, 16.9 %): ^1^H NMR (400 MHz, [D_6_]DMSO): *δ*=7.78 (dt, 1 H), 7.73 (t, 1 H), 7.55 (t, 1 H), 7.50 (dd, 2 H), 7.43 (m, 3 H), 7.35 (tt, 1 H), 6.60 (br s, 2 H), 5.20 ppm (s, 2 H); MS (ESI): *m*/*z*=296 [*M*+H]^+^.

1 m BCl_3_ in CH_2_Cl_2_ (5.22 mL, 5.22 mmol) was added dropwise to a stirred solution of (4-aminofurazan-3-yl)-(3-benzyloxyphenyl)methanone (770 mg, 2.61 mmol) in CH_2_Cl_2_ (20 mL), and the mixture was stirred at RT for 30 min. MeOH (1 mL) was added, the mixture was concentrated to dryness in vacuo, and the residue purified by column chromatography on silica gel (EtOAc/petroleum ether, 0–40 %) to give (4-aminofurazan-3-yl)-(3-hydroxyphenyl)methanone as a pale tan solid (500 mg, 93 %): ^1^H NMR (400 MHz, [D_6_]DMSO): *δ*=9.97 (s, 1 H), 7.62 (dm, 1 H), 7.56 (m, 1 H), 7.42 (t, 1 H), 7.15 (dm, 1 H), 6.57 ppm (br s, 2 H).

NaH (60 wt % in mineral oil, 180 mg, 4.58 mmol) was added to a mixture of (4-aminofurazan-3-yl)-(3-hydroxyphenyl)methanone (250 mg, 1.22 mmol) and ethyl bromoacetate (0.42 mL, 3.66 mmol) in DMF (10 mL), and the mixture was stirred at 75 °C for 2 h. The reaction mixture was allowed to cool to RT and then partitioned between EtOAc and water. The organic layer was washed with water and then brine, separated, dried over MgSO_4_, filtered, and concentrated in vacuo. The residue was purified by column chromatography on silica gel (EtOAc/petroleum ether, 0–30 %) to afford semi-pure product, which was further purified by preparative HPLC to afford **7** as a colourless solid (23 mg, 6.5 %): ^1^H NMR (400 MHz, [D_6_]DMSO): *δ*=7.80 (dt, 1 H), 7.65 (t, 1 H), 7.55 (t, 1 H), 7.35 (dt, 1 H), 6.60 (br s, 2 H), 4.90 (s, 2 H), 4.20 (q, 2 H), 1.23 ppm (t, 3 H); MS (ESI): *m*/*z*=292 [*M*+H]^+^.

**(4-Aminofurazan-3-yl)-[3-(1*H*-benzoimidazol-2-ylmethoxy)phenyl]methanone (8)**: NaH (60 wt % in mineral oil, 29 mg, 0.73 mmol) was added to a mixture of (4-aminofurazan-3-yl)-(3-hydroxyphenyl)methanone [prepared as outlined above for the synthesis of **7**] (75 mg, 0.37 mmol) and 2-chloromethyl-1*H*-benzimidazole (122 mg, 0.73 mmol) in DMF (10 mL), and the mixture was stirred at 75 °C overnight. The reaction mixture was allowed to cool to RT and was then partitioned between EtOAc and water. The organic layer was washed twice with water, separated, dried over anhydrous MgSO_4_, filtered and concentrated in vacuo. The residue was purified by preparative HPLC to afford **8** as a colourless solid (5 mg, 4.1 %): ^1^H NMR (400 MHz, [D_6_]DMSO): *δ*=12.70 (br s, 1 H), 7.80 (m, 2 H), 7.63 (dd, 1 H), 7.58 (t, 1 H), 7.50 (td, 2 H), 7.20 (m, 2 H), 6.60 (br s, 2 H), 5.43 ppm (br s, 2 H); MS (ESI): *m*/*z*=336 [*M*+H]^+^.

## Author Contributions

S.M.S-B. designed the protein constructs, and expressed, purified, characterized and crystallized the protein. C. W. M. analysed the PDB for comparative HCO_3_^−^ binding interactions. V.B. modelled and designed the compounds and produced the pictures. A.C. performed crystallization, X-ray data collection, structure solution by multiple isomorphous replacement, and solution of the AMPCPP and bicarbonate complexes. M.V. performed ligand soaking experiments, X-ray data collection, solved and refined the complex structures, measured the p*K*_a_ values of compounds, searched compound databases for hit enrichment, and designed compounds. M.C. and M.A.O’B designed and synthesized compounds. J.E.C performed the *T*_m_ shift screen. H.J. and J.R.Y. helped define the allosteric model and manage the project. S.M.S-B and J.E.C. were project leaders during structure solution and pyramid screening phases, respectively. S.M.S-B., C.W.M. and H.J. wrote the manuscript.

## Supporting Information

As a service to our authors and readers, this journal provides supporting information supplied by the authors. Such materials are peer reviewed and may be re-organized for online delivery, but are not copy-edited or typeset. Technical support issues arising from supporting information (other than missing files) should be addressed to the authors.

miscellaneous_information
