# Supplementary material for: Crystal Structure of Human Soluble Adenylate Cyclase Reveals a Distinct, Highly Flexible Allosteric Bicarbonate Binding Pocket
Source: ChemMedChem. 2014 Feb 24;9(4):823–32. doi: 10.1002/cmdc.201300480 (PMC4506562; doi:10.1002/cmdc.201300480)
Supplement: Supplementary file 1 — miscellaneous_information [file cmdc0009-0823-sd1.pdf]

CHEMMEDCHEM
Chemistry & Drug Discovery

Supporting Information

© Copyright Wiley-VCH Verlag GmbH & Co. KGaA, 69451 Weinheim, 2014

Crystal Structure of Human Soluble Adenylate Cyclase Reveals a Distinct, Highly Flexible Allosteric Bicarbonate Binding Pocket

Susanne M. Saalau-Bethell, Valerio Berdini, Anne Cleasby, Miles Congreve, Joseph E. Coyle, Victoria Lock, Christopher W. Murray, M. Alistair O'Brien, Sharna J. Rich, Tracey Sambrook, Mladen Vinkovic, Jeff R. Yon, and Harren Jhoti*[a]

cmdc_201300480_sm_miscellaneous_information.pdf

Supplementary Information

Regulation of hsolAC by bicarbonate

The effects of bicarbonate on mammalian solAC's have been shown to be two-fold: firstly, it relieves substrate inhibition and secondly it increases V_{max}. The latter effect could be achieved by either altering the effective concentration of protein in a productive catalytic state [E_0] or by altering the rate constant (k_{cat}). The rate limiting step for the conversion of ATP to cAMP by hsolAC has not yet been determined, but in tmAC's is reported to be product release. [32]

Substrate inhibition of hsolAC at high ATP concentrations suggest that product release might be rate limiting, since it would lead to a build-up of enzyme-product complex [E-P] and a concomitant decrease in catalytically competent enzyme concentration [E_0]. It is possible that the effect of HCO_3^- on hsolAC catalysis might be exerted by expediting the release of product, leading to an increase in [E_0] and an associated increase in V_{max} (V_{max}=k_{cat}[E_0]). This might also explain the relief of substrate inhibition at high concentrations. The crystal structure of bicarbonate bound hsolAC suggests that Arg176 (and Lys95) plays a key role in the activation of the enzyme, however in the absence of the ternary complex structure (AMPCPP/$Ca^{2+/}HCO_3^-$), the substantial movements of the loops around the active site upon ligand binding and difference in sequences across species it would be hard to speculate what other residues are likely to be involved. Work on the cyanobacterial *Anabaena sp.* PCC7120 showed that Lys646 (Lys95 in hsolAC) coordinated the bicarbonate ion and proposed the involvement of a conserved threonine residue (Thr721) in bicarbonate responsiveness. [20] Our structures however show that the corresponding threonine residue (Thr405) is only present in C2 and is located in the nucleotide binding site as opposed to the allosteric bicarbonate site.

Comparison between in hsolAC and S. platensis

The structure of hsolAC was compared with the previously solved structure of *Spirulina platensis* adenylate cyclase (PDB code 1wc1) [17] using Comparer [33] and MNYFIT.[34] In order to minimise differences due to large domain movements, the comparison was done for the two individual domains, the first domain pair having an rmsd of 1.2Å (116 out of 181 residues being designated as equivalent), and the second domain pair having an rmsd of 1.4Å (127 out of 179 residues).

Significant differences were reported in the binding of AMPCPP (α,β-Me-ATP) and Rp-ATPαS to *S. platensis* solAC [17] resulting and productive and non-productive binding modes. For the purpose of comparing the interactions between the protein and the nucleotide, we looked at the binding of AMPCPP to both enzymes and observed that the nucleotides overlap relatively well, though the position of the β and γ Phosphates are different. Main differences are the positioning of the catalytic NxxxR groups (Asn412 and Arg416 in hsolAC and Asn1146 and Arg1150 in the bacterial enzyme), which appear better engaged in the hsolAC structure. The Asn side chains are in very similar position, but twisted such that in hsolAC the $N_{\delta2}$ interacts with an oxygen from the γ Phosphate, while the $O_{\delta1}$ interacts with the base. In the *S. platensis* complex structure, Asn1146 only appears to form water mediated interactions to the nucleotide. The Arg side chains display different orientations starting at the Cδ position at an angle of about 80°. The *S. platensis* structure does not appear to directly interact with the nucleotide, but forms water mediated H-bonds, whilst the guanidino group of Arg416 in hsolAC structure H-bonds to the α Phosphate.

Fragment screen

A library of~ 1600 fragments was screened by thermal shift. Fragments giving a ΔTm of ≥ 0.5°C were classed as hits; overall, thermal shift identified 31 hits corresponding to a ~ 2% hit rate. A subset of the fragment library (441 fragments) was also screened using the bioassay, of which 21 fragments gave >40% enzyme inhibition at 1mM and were classed as hits, equivalent to a ~ 5% hit rate. Thermal shift identified 11 hits within the same 441 fragment subset. Within this subset, 6 hits were unique to thermal shift, 16 hits were unique to bioassay and 5 hits were observed by both methods. Informatively, the 5 hits detected in both thermal shift and bioassay screens were successfully visualised by X-ray. Overall, 46 hits from the thermal shift and bioassay screen were progressed into X-ray soaks and 12 fragments were observed binding within the enzyme active site.

Supplementary table 1

Compound	Conc [mM]	Soak time	No of sites (Major/minor)	Resolution /Angstrom	Phasing power[a]
thimersal	0.05	2.5hours	4	2.5	0.98
Potassium iodide	150	2 min	2	2.5	0.62
Dichloroethylene diamine platinum	Saturated	5.5 hours	1(3)	2.2	0.57
Ammonium tetrabromoosmate	1	3 days	4	2.8	0.66
Trimethyl lead acetate	10	3 days	3	3.0	0.89

Supplementary table 1: Soaking conditions and data quality for the heavy atom derivatives used to solve the structure of hsolAC

[a]Phasing power = mean heavy-atom amplitude / mean *P*-weighted lack of closure error

Supplementary table 2a: Diffraction Data and Structure Refinement Statistics

	**Apo**	**AMPCPP**	**Bicarbonate**	**Cpd 1**	**Cpd 2**
Data collection					
**Collection site**	ESRF ID29	Rigaku RUH/Raxis	ESRF ID23-1	ESRF ID14-2	ESRF ID29
**Space group**	$P6_3$	$P6_3$	$P6_3$	$P6_3$	$P6_3$

**Unit cell: a, c/Å**	99.4, 97.4	99.7, 96.5	99.8, 98.2	99.4, 97.6	99.3, 97.6
**Resolution/Å**	49.7-1.7	39.4-1.89	27.6-1.74	27.5-2.1	28.7-2.28
**Rmerge/%**	10.5	4.3	6.2	8.5	8.7
**I/sigI outer shell**	1.4	2.4	2.2	1.7	1.2
**Completeness/%**	99.8	65.7(95.7 to 2.4Å)	98.5	96.5	96.1
**Redundancy**	3.6	3.0	2.4	2.6	2.9
Refinement					
**R/Rfree(/%)**[1]	18.2/21.1	16.8/23.0	16.7/20.3	16.6/22.1	16.4/22.8
**B-factor(all non water atoms)**	31.3	50.2	31.3	33.9	38.1
**B-factor (ligand)**	-	50.0	23.3	36.3	49.0
**Rmsd bonds/Å**	0.013	0.012	0.013	0.012	0.011
**Rmsd angles/°**	1.1	1.2	1.1	1.1	1.1

[1] Rfree is calculated for 5% randomly chosen reflection not included in the refinement. The same reflections were used for all data sets.

Supplementary table 2b

	**Cpd 3**	**Cpd 4**	**Cpd 5**	**Cpd 6**	**Cpd 7**	**Cpd 8**
Data collection						
**Collection site**	ESRF ID29	ESRF ID29	ESRF ID29	ESRF ID23-1	ESRF ID29	ESRF ID29
**Spacegroup**	$P6_3$	$P6_3$	$P6_3$	$P6_3$	$P6_3$	$P6_3$
**Unit cell: a,c/Å**	100.0, 98.0	99.0, 97.7	99.3, 97.6	99.3, 98.0	99.7, 98.1	99.7, 98.2
**Resolution**	28.9-1.75	28.6-1.7	27.2-1.7	27.5-1.7	28.8-1.7	28.7-2
**Rmerge**	6.6	4.7	7.2	4.8	6.1	7
**I/sigI outer shell**	2.3	2.9	3.2	2.4	2.0	2.5
**Completeness**	99.7	95.1	94.4	94.9	99.4	99.6
**Redundancy**	2.8	2.6	2.6	2.2	2.7	2.6
Refinement						
**R/Rfree**[1]	17.6/21.2	17.4/20.8	17.9/21.6	17.0/21.6	17.2/21.1	16.1/21.0
**B-factor(all non water atoms)**	29.8	33.2	31.8	36.0	28.9	30.1
**B-factor (ligand)**	35.2 (1)<br>28.2 (2)<br>33.8 (3)	29.0	38.6	40.2	28.1	43.1
**Rmsd (bonds)**	0.013	0.011	0.012	0.011	0.011	0.012
**Rmsd (angles)**	1.2	1.5	1.5	1.5	1.5	1.1

[1] Rfree is calculated for 5% randomly chosen reflection not included in the refinement. The same reflections were used for all data sets.

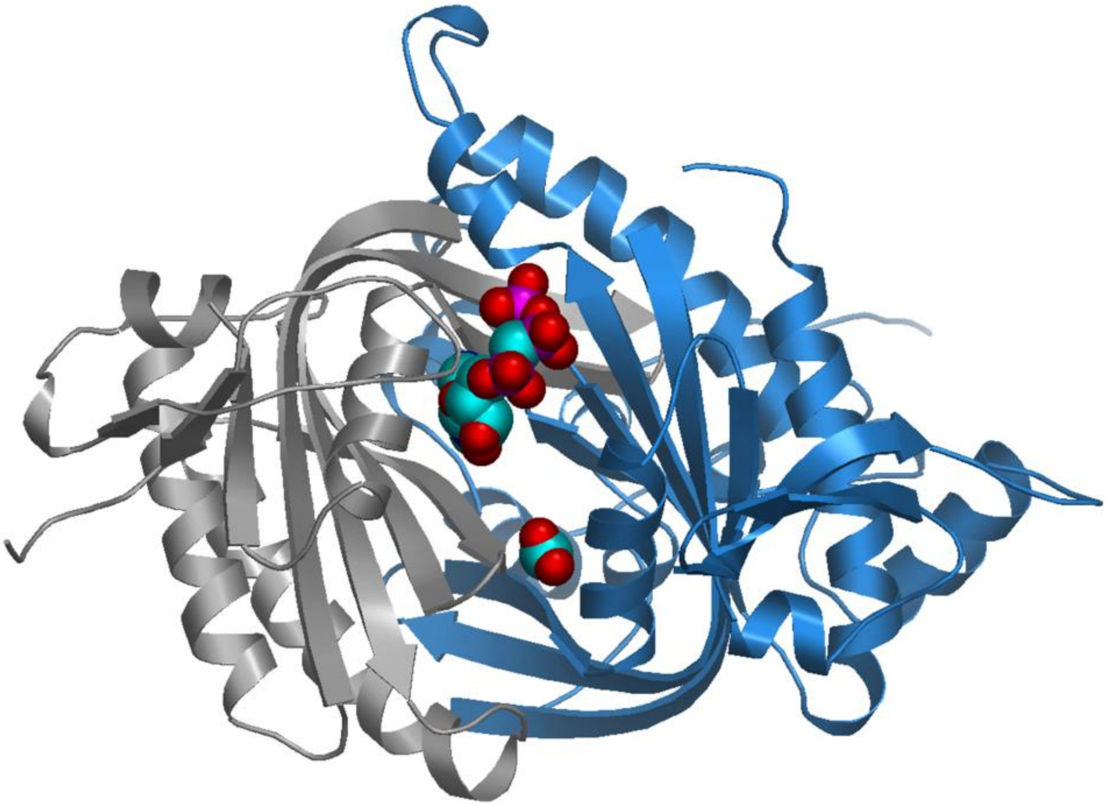

Supplementary Figure 1: Crystal Structure of the human adenylate cyclase
Ribbon diagram of human soluble adenylate cyclase enzyme in complex with the substrate analogue AMPCPP. Superimposed on this structure is the crystallographically determined binding location of the bicarbonate ion. Both molecules are shown as solid spheres. Domains C1 and C2 of the single chain are coloured blue and grey, respectively, and show the 2-fold pseudo-symmetry of the protein.

```
                                      α1         α2             β1          α3
hsolAC    C1       ------MNTPKEEFQDWP-IVRIAAHLPDLIVYGHFSPERPFMDYFDGVLMFVDISGFTA
hsolAC    C2       LACTLKPDPELEMSLQKYVMESILKQIDNKQLQGYLSELRPVTIVFVN-LMFEDQDKAEE
S.platensis        --------------------------MGDLTLD--LRPEPRLIT-----ILFSDIVGFTR
                                                             β1              α1

                                      α4                   β2      β3
hsolAC    C1       MTEKFSSAMYMDRGAEQLVEILNYHISAIVEKVLIFGGDILKFAGDALLALWRVERKQ--
hsolAC    C2       IGPAIQD------AYMHITSVLKIFQ-----------GQINKVFMFDKGCSFLCVFGFPG
S.platensis        MSNALQ--------SQGVAELLNEYLGEMTRAVFENQGTVDKFVGDAIMALYGAPEEMSP
                                      α2                   β2      β3

                       α5                                       β4
hsolAC    C1       LKNIITVVIKCSLEIHGLFET-QEWEEG--------LDIRVKIGLAAGHISMLVFGDETH
hsolAC    C2       EKVPDELTHALECAMDIFDFCSQVHKIQTVS-----------IGVASGIVFCGIVGHTVR
S.platensis        SEQVRRAIATARQMLVALEKLNQGWQERGLVGRNEVPPVRFRCGIHQGMAVVGLFGSQER
                       α3                                       β4

                     β5        α6           β6   α7         β7              β8
hsolAC    C1       SHFLVIGQAVDDVRLAQNMAQMNDVILSPNCWQLCDRSMIEIESVPDQ------RAVKVN
hsolAC    C2       HEYTVIGQKVNLAARMMMYYPG-IVTCDSVTYNGSNLPAYFFKELPKKVMKGVADSGPLY
S.platensis        SDFTAIGPSVNIAARLQEATAPNSIMVSAMVAQYVPD—EEIIKREFLELKGIDEPVMTC
                     β5        α4          β6    α5          β7             β8

                               α8              α9
hsolAC    C1       FLKPPPNFNFDEFFTKCTTFMHYYPSGEHKNLLR-
hsolAC    C2       QYWGRTEKV--------------------------
S.platensis        VINPNMLNQ--------------------------
```

Supplementary figure 2: Alignment of hsolAC and *Spirulina platensis* solAC. Highlighted in red are the α- helices, in light blue the β-strands. The nomenclature in bold over the alignment refers to the C1 and C2 of the human enzyme, whilst that under the alignment and italised refers to the *S.platensis* structure.
